# From Copyright to Copyleft to CopyCovid: An opportunity for democratization of medical education

**DOI:** 10.15694/mep.2021.000025.1

**Published:** 2021-01-28

**Authors:** Vildan Ozeke, Işıl İrem Budakoğlu, Yavuz Selim Kıyak, Özlem Coşkun

**Affiliations:** 1 Tokat Gaziosmanpasa Universitesi; 2 Gazi Universitesi

**Keywords:** COVID-19, pandemic, democratization of medical education, open-access, online learning

## Abstract

This article was migrated. The article was not marked as recommended.

The COVID-19 (Coronavirus Disease of 2019) pandemic, as modern world’s gravest health crisis, has rapidly required more health facilities and health care providers (HCPs). Education system in general and medical education in particular has also changed dramatically with the school closures. Teaching and learning activities are undertaken remotely and on digital platforms. Academicians, HCPs, and medical students has gained new skills and experienced to manage their own learning process online. Many sources became easily reachable, open and free. Open and democratized medicine, as we seen during this pandemic, offers an interactive, egalitarian educational model. Governments, global organizations and companies collaborated in order to keep all students learning and all instructors teaching. Many societies faced with digital divide and exclusion of learners. In this paper we discussed the effect of COVID-19 on medical education at all levels and its’ democratization, and shared our recommendations for sustaining of these achievements after the pandemic.

## What is happening?

The COVID-19 pandemic, as modern world’s gravest health crisis, has rapidly required more health facilities and health care providers (HCPs). In some countries the health systems were inadequate and became unable to perform efficiently in terms of COVID-19 challenges such as “ *the number and spread of cases, the social, economic and healthcare capacity to manage disruption*” ( Tolsgaard *et al.,* 2020). After people realized the fact that the virus is spreading by contact and micro-particles in the air caused daily life routines and all social dynamics to blow up. We all migrated to the digital world. For the Organization for Economic Co-operation and Development (OECD), the COVID-19 crisis not only threatens human health but also stagnates human capital ( Williamson, 2020). Education system in general and medical education in particular have changed dramatically. The United Nations Educational, Scientific and Cultural Organization (UNESCO) announced the COVID-19 outbreak as a major education crisis. Teaching and learning activities are undertaken remotely and on digital platforms. In-person courses transformed into virtual courses, clinical rotations have been suspended, the clinical responsibilities and educational opportunities of residents have been altered ( Woolliscroft, 2020). In another perspective, the COVID-19 outbreak provided big chance for education technology companies in order to show what they can do, and most of them made their resources freely available. The shareholders had an opportunity for experimentation of technology supported learning environments. The videoconferencing platforms (Zoom, Microsoft Teams, Google Classroom/Meet), learning management systems, and cloud-based education services were widely used around the world to collaborate and to set/to perform remote learning tasks ( Williamson, 2020). Many selected digital resources, archives, and museums became open to public during lockdowns (Audible by Amazon, British Museum, The Museum of Modern Art, The Metropolitan Opera, and The Berlin Philharmonic etc.). Some libraries, databases and publishing companies (e.g. Cambridge University Press, EBSCO, Elsevier, JSTOR, Sage Publishing, Springer, Wiley, and Wolters Kluwer) allowed free access (mostly related to COVID-19, pandemics, and virus) to their databases for this uncertain period. Medical College Admission Test (MCAT) Collection became freely available on the Khan Academy, sponsored by The Association of American Medical Colleges (AAMC).

During the coronavirus pandemic, as part of synchronous communication the video/web-conferencing tools, webinars has become popular to manage meetings ( Macgilchrist, 2020). AMEE-hosted a webinar that focused on the impact of the pandemic across the continuum of medical education and training ( Cleland *et al.,* 2020). Many academicians have organized webinars to contact and help medical students and fellows and to continue education over this unprecedented period as part of “Emergency Remote Teaching” ( Hodges *et al.,* 2020). These meetings have catalyzed free and interactive education with few exceptions (i.e. need a password to be able to participate in the meetings). It provided an opportunity for physicians, fellows and medical students from all around the world to join and listen to unique lessons by well-known academicians and physicians ( Almarzooq, Lopes and Kochar, 2020). In terms of undergraduate medical education, it was a chaotic process with a lot of trial and error involved. The high number of students (greater than 300) in a class made it impossible to join synchronous sessions. On the other hand dividing the classrooms into manageable sizes and conducting synchronous sessions increased the workload of instructors. Problem based learning and case based learning sessions carried out with small groups through webinars. Unfortunately the clinical clerkships of undergraduates were cancelled and medical students were pulled out of clinical environments for infection control purposes ( Tolsgaard *et al.,* 2020).

## Where we were before the pandemic?

Indeed, the wide and freely accessible information has always been restricted. The cost of accessing medical content still remains a challenge in developing countries, and many physicians cannot afford access to updated medical information ( Gibson, 2017). The inequality of the distribution of educational resources exists not only between countries but also within countries ( Acemoglu, Laibson and List, 2014). Although the internet has decreased the cost of sharing and storing information, the barriers of expensive or closed distribution of educational content remain as an issue ( Ozeke *et al.,* 2020). At this stage, there are some efforts on the understanding of this ignored problem. Acemoglu *et al.* (2014) emphasize the major impact of web-based educational technologies as the democratization of education. Everybody benefits from the educational resources which are distributed more equally. WikiDoc platform, a nonprofit foundation, launched by Dr. Gibson in late 2005 as the world’s largest medical textbook, allows people all over the world to access the same high level of advanced medical information. He also developed http://www.clinicaltrialresults.org as a website to share PowerPoint slides among physicians as part of the open-access (OA) movement and gave several presentations about the importance of the democratization of medical knowledge and social media on medical education ( Gibson, 2014; Gibson, 2017; Gibson, 2020). Free Open Access Meducation (FOAM) movement ( Cadogan *et al.,* 2014) is another free accessible collection that holds blogs, podcasts, Google Hangout sessions, online videos, text-based documents, images, social media posts (tweets, Facebook groups) etc. about medical topics. Health 2.0 or Medicine 2.0 has shifted from displaying static webpages with a one-way direction of information flow to participatory communities with bidirectional flow of information ( Gibson, 2017; Gibson, 2020) between HCPs, academicians, medical students, and patients. The Human Diagnosis Project (Human Dx) aims to join medical professionals and trainees in a coordinated way for building a collective intelligence with machine learning.

Massive Open Online Courses (MOOCs) (e.g. Coursera, Khan Academy) are the result of OA course materials initiative of some big universities in USA. Khan Academy’s mission is providing the same unified sources to learners and support them to participate in a free global education ( Williamson, 2020). The MOOCs aim to present free access to the course materials (pre-recorded videos, online discussion boards, assessment tools (test, exam, quizzes)) to the huge numbers of students from all around the world. Some of these courses don’t cost any money, and even they offer free printable certificates when completed.

Each step towards the democratization of knowledge not only contribute to the correction of social inequalities but also creates an opportunity for HCPs, academic physicians, residents and medical students to stay updated on latest medical topics and to bridge disciplines ( Bondarenko and Kozulin, 1991; Colt and Quadrelli, 2006). Some academicians in Europe announced the ‘Plan S’ as a radical OA initiative to make all scientific works free to read as soon as they are published ( Else, 2018). The benefit of OA is its global impact and broader audience. Recent policies tackle OA philosophy as an urgent priority in order to maximize these benefits for science, society, and future.

## Time changes things: From Copyright to Copyleft to CopyCovid

Indeed, it is an interesting story of the origin of the term “copyright”. After the printing press came into use in Europe, it allowed the rapid distribution of large volumes of printed work, some of which could be critical of English government ( Gibson, 2020; Wikipedia, 2020b). In reaction to the printing of “scandalous books and pamphlets”, the English Parliament passed the Licensing of the Press Act 1662. According to the act, all intended publications required to be registered with the government-approved company which has the right to allow which material could be printed ( Wikipedia, 2020b). The King permitted only certain people the “Right to Copy” work, therefore, the original copyright law was actually a censorship law from the beginning of the printing press ( Wikipedia, 2020b; Gibson, 2020). The establishment of copyright law in 1710s England caused the low-price mass printing market vanished, and fewer, more expensive editions were published ( Wikipedia, 2020b). Publishing was the only way to reach a wider audience in those days. Scientific and technical information was greatly reduced due to accessing and distribution issues ( Wikipedia, 2020b). A rigid paternalistic educational model was dominating science.

The term of “Copyleft” was first described in 1985 by a programmer who is free software movement activist ( Wikipedia, 2020a). The Copyleft is offering people the right to freely use, modify, copy, and distribute a work and modified versions of it with the stipulation that the same rights be preserved in derivative works created later ( Wikipedia, 2020a). In the digital world, dissemination of information is very rapid and information sources have a high degree of diversity. Nobody wants to start from zero which is seen as a waste of time. Copyleft licensing is another way of collaborating as a win-win situation. The copyleft philosophy forms a basis for Dr. Gibson’s attempts in terms of democratization of medical education ( Gibson, 2017; Gibson, 2020).

Open and democratized medical knowledge, as we seen during this pandemic, offers an interactive, egalitarian educational model ( Colt and Quadrelli, 2006). We may call this case as “CopyCovid” in particular and “Copydemic” in general. Although the COVID-19 pandemic triggered all these interactions, we can also use “Copydemic”, because it is more inclusive. In the CopyCovid/Copydemic times, “ *all the red tape that keeps things away is gone and people are looking for solutions that in the past they did not want to see*” says Andreas Schleicher who is the head of the education division at the OECD ( Schleicher, 2020). There were many OA digital resource initiatives in Medicine 2.0 for more than a decade, however COVID-19 is a milestone that has broken taboos on teaching and learning in digital environments. People are interacting and communicating with technology at each moment. Nowadays most of all academicians, HCPs, and medical students gained new skills and experienced to manage their own learning process online. Furthermore, especially medical academics are under the influence of attractive informal processes; they are continuing lectures, joining courses outside of their formal education in the context of continuous medical education. The global pandemic may be seen as an experimental opportunity to set the stage for long-term digital transformations in education system ( Williamson, 2020). Because of the pandemic, nearly all of the annual meetings are shifted towards organizing completely as virtual events. AMEE 2020 will be held virtually for the first time of its 20 year history. It means that without accommodation and transportation fees it will be more affordable for low-income academics.

In the CopyCovid/Copydemic times, the international organizations (Global Partnership for Education, OECD, UNESCO, WHO, and World Bank) and some companies (e.g. Google, Microsoft, Zoom) made a coalition not only as a short term support of sustaining education but also as a long term investing for future education ( Williamson, 2020). School closures showed again the fact that various inequities in accessing technology exist all around the world ( Cleland *et al.,* 2020). This alliance, COVID-19 Education Coalition, aimed never to stop learning and to maintain inclusion of each student no matter what their socio-economic status is. For example, Google’s hub of information and tools called as “Teach from Home”, which is consisting of the standard Google G Suite of apps for education, is launched in partnership with UNESCO’s Institute for Information Technologies in Education and the International Society for Technology in Education (ISTE) in order to help instructors and learners during the pandemic.

Open-source and free Learning Management Systems (LMS) are featured for interacting during the pandemic in many universities. However main issue was the Internet quota limits of users. In Turkey, The Council of Higher Education (CoHE) made an agreement with GSM operators to add extra 6 GB quota while using pre-determined distance education sources. However medical education courses are not included in these pre-determined digital sources. Our university made a protocol with GSM operators to add extra 8-10 GB quota for supporting especially low-income students. Although students need additional quotas for the Internet (if they don’t have free Wi-Fi), they economize paying on textbooks and other nonweb resources ( Acemoglu, Laibson and List, 2014).

The CopyCovid/Copydemic times pushed medical educators to rapidly convert their in-person didactic lectures and tutorials to virtual courses ( Woolliscroft, 2020) or using existing videos and lectures on the Internet. However, these interactions still need to be complemented with one-on-one discussions provided by local educators ( Acemoglu, Laibson and List, 2014). This situation forced educators to innovate and think out of the box to maintain quality medical education ( Liang, Ooi and Wang, 2020). Among the social media platforms, Twitter has been important part of disseminating scientific and empirical knowledge between physicians, HCPs, and medical academics to exchange ideas, tools, learning activities and to follow webinars and new sources. The COVID-19 crisis, is a story of collaboration and social interaction while social distancing ( Macgilchrist, 2020; Cleland *et al.,* 2020).

Although it uses the sources of distance education, the term of “emergency remote teaching (ERT)” is the best way to define what teachers, instructors and academics are trying to achieve. The dynamics of ERT are very different from usual distance/online education in terms of its philosophy. The ERT is trying to translate familiar forms of face-to-face teaching into remote learning by using online education tools ( Macgilchrist, 2020; Hodges *et al.,* 2020). Technology‐mediated remote teaching has so many platforms between unstructured to well-structured. At this stage, instructional design specialists are giving us a warning about the focus should be on learning rather than on technology or tools ( Spector, 2020). Interdisciplinary collaborations are needed more than ever before to design and develop well-structured learning environments. Because the new forms of teaching and training (e.g. e-learning, online learning, flipped learning, emergency remote learning) require new instructional approaches, strategies, methods and evaluation metrics. The affective, social and cognitive aspects of teaching, learning and assessment should be considered by mixing and varying synchronous and asynchronous tools while connecting with medical students as independent learners.

## What we need to sustain democratization of medical education after the pandemic?

We will not know the full impact of COVID-19 on medical education ( Ferrel and Ryan, 2020), the horizon is not clear enough. Nevertheless, the earned gains, such as digital literacy, self-regulation, collaboration etc., during the pandemic hopefully will continue to move forward on the path of democratization of medical knowledge. When the pandemic completely ends, we should not simply return to our teaching and learning practices and habits before the COVID-19 ( Almarzooq, Lopes and Kochar, 2020; Hodges *et al.,* 2020). The societies realized that this pandemic is first in digital era, but not to be the last, so countries should be ready for another serious outbreak. The coalitions are developing new long-term policy plans for future education systems worldwide ( Williamson, 2020). The practices we interact with today are just a demonstration, first-draft outline of existing emergency policy developments that are still on the move ( Williamson, 2020). Best practices should be shared worldwide in order to improve performances of all participants. For example, the“Learning Keeps Going” portal ( https://www.learningkeepsgoing.org) is a good initiative to support communities with help desk, webinars, podcasts and many other ways about online learning during school closures.

Medical educators around the world may form their “communities of practice” in order to engage in a process of collective learning. So they will teach themselves while trying to teach the students effectively. All actors in the environment related to medical knowledge can be called as, “community of practice” ( Lave and Wenger, 1991). To let novices to go through their identity journey and to let them arrive their target on the way of legitimate peripheral participation, we may choose the way we build “a Rawlsian system of cooperation” among community of practice. As Rawls’ Theory of Justice proposed ( Lovett, 2010), we need to determine basic structure of our community. Rawls defines it as “the way in which the major social institutions distribute fundamental rights and duties and determine the division of advantages from social cooperation” ( Lovett, 2010). What we have seen during CopyCovid/Copydemic times may be first global footsteps of the egalitarian distribution of fundamental rights such as reaching education to strengthen cooperation. In order to democratize the medical education, distribution of information, courses, digital sources, and tools need to be more freely than it is currently.

Digital divide, not having related technology or insufficient access to digital sources, cause some students to exclude from learning. Universities, public libraries, schools should offer solutions by opening/sharing their facilities and/or Internet connections free to these students. On the other hand, medical academics are testing out different digital learning environments and tools may be for the first time. Universities should empower instructors in terms of using these tools, provide help desks in designing and developing course contents and encourage them to think creatively ( Williamson, 2020).

Governments and scientific foundations should spend more on educational technology projects and support all kind of initiatives to develop tools, simulations and learning environments especially on medical education. The clerkships need to be transformed into virtual learning environments with augmented reality or simulation technologies.

In conclusion, digital transformation, which started a long time ago in medical education, gained a great momentum with the CopyCovid/Copydemic times and provided equality and democratization for students and academicians in terms of accessing to the valid information. The industry providing services in this field has put their profit anxiety on the second plan in the difficult days of the world, and the main reason for their existence - giving priority to providing information- has come to the fore in service delivery. It is anticipated that students, academics and administrators who gain knowledge and skills on accessing to the right information sources, critical thinking, designing/developing e-learning materials and self-learning will restructure medical education in the following years and nothing will be the same.

## Take Home Messages

• COVID-19 pandemic has transformed medical education into digital platforms.
• The lockdowns gave an opportunity for democratization of medical education in terms of open access sources, free online courses etc.
• “CopyCovid/Copydemic” term, which is transformed from Copyright/Copyleft, has been born during the pandemic.
• In order to sustain the achievements after the pandemic, some recommendations were shared.

## Notes On Contributors

**Vildan Ozeke** is an Assoc. Proffesor in Tokat Gaziosmanpasa University, Department of Educational Sciences, Curriculum and Instruction in Tokat, Turkey. She also studies as a postdoctoral researcher in Gazi University, Department of Medical Education and Informatics, Ankara, Turkey. Her areas of interests include instructional design, ICT integration in medical education. ORCID ID: https://orcid.org/0000-0003-1827-1258

**Isil Irem Budakoglu** is a Proffessor in Gazi University, Department of Medical Education and Informatics, Ankara, Turkey. She is Head of the Department since 2011. Her areas of interests include clinical leadership and management, healthcare professionals and communication skills, medical education at all levels, curriculum development and problem-based learning. ORCID ID: https://orcid.org/0000-0003-1517-3169

**Yavuz Selim Kiyak** is a faculty member of Gazi University Faculty of Medicine, Department of Medical Education and Informatics. He is also PhD student at Medical Education. He dedicated himself to spread the information regarding Medical Education. ORCID ID: https://orcid.org/0000-0002-5026-3234

**Ozlem Coskun** is an Assoc. Proffesor in Gazi University, Department of Medical Education and Informatics, Ankara, Turkey. Her areas of interests include medical education (face-to-face and e-learning), instructional design, curriculum development, rational drug use, evidence-based medicine, leadership and crisis management, mentoring, coaching, and consulting in medical education. ORCID ID: https://orcid.org/0000-0001-8716-1584
