# Supplementary material for: From Copyright to Copyleft to CopyCovid: An opportunity for democratization of medical education
Source: MedEdPublish (2016). 2021 Sep 21;10:25. Originally published 2021 Jan 28. [Version 2] doi: 10.15694/mep.2021.000025.2 (PMC10939593; doi:10.15694/mep.2021.000025.2)
Supplement: Peer review history

# Reviewer report: Reviewer response for version 2

**DOI:** 10.21956/mep.20209.r31288

**Published:** 2021-10-05

**Authors:** Julie Hunt

**Affiliations:** 1 Lincoln Memorial University

This review has been migrated. The reviewer awarded 4 stars out of 5 The authors describe the changes to copywrite practices that have occurred recently as a result of the COVID-19 pandemic, offer their opinions on the suitability of these changes for medical education and in particular in resource-constrained environments and for resource-constrained people. The authors also offer insight as to where they see the field of medical education going with respect to open access and democratization.I found figure 1 helpful in understanding the features of copywrite, copyleft, and copycovid as defined by the authors. Overall I think that this article would be of interest to those in the field who are openly sharing educational resources, or those who are re-considering whether to openly share these resources as the pandemic (eventually) draws to a close.

Reviewer Expertise:

NA

No decision status is available

# Reviewer report: Reviewer response for version 2

**DOI:** 10.21956/mep.20209.r31289

**Published:** 2021-10-03

**Authors:** Ken Masters

**Affiliations:** 1 Sultan Qaboos University

This review has been migrated. The reviewer awarded 4 stars out of 5 I am pleased to see that the authors have addressed all of my major concerns related to Version 1 of the paper, and have produced a paper that is much easier to read, follow and understand. Although some readers may take issue with some of the arguments presented, it is a worthwhile contribution to the field.The full impact of Covid on the issue of copyright still has some time to play out, and this paper will allow people to understand the developments surrounding the issues.

Reviewer Expertise:

NA

No decision status is available

# Reviewer report: Reviewer response for version 1

**DOI:** 10.21956/mep.19014.r27287

**Published:** 2021-02-04

**Authors:** Ken Masters

**Affiliations:** 1 Sultan Qaboos University

This review has been migrated. The reviewer awarded 2 stars out of 5 The paper discusses the impact of COVID-19 on copyright issues and the resultant implications for medical education.Overall, the authors do make several valid points regarding changes and the potential for change, but the paper suffers from several problems that need to be addressed.• Many of the paragraphs consist of a listing of events. It would help the authors if they broke some of their longer paragraphs into smaller pieces, and more directly used the events to develop an argument throughout the paper. Often the events are listed, with little indication of the connections, relationships or their role in developing the argument. Having sub-headings may help the process.• Although it is true to say that many of the academic publishing houses allowed access to some of their material, one should also be somewhat sceptical of this, as it does not indicate any change of heart. This was a very temporary, specific and targeted exercise, accompanied (motivated?) by a great deal of self-advertising, and, no doubt, commercial expediency. It should be noted that academic publishing houses have repeatedly and successfully sued Sci-Hub for copyright infringement, and have not reduced their efforts during the COVID-19 crisis. In fact, right now, Elsevier, Wiley and the American Chemical Society are suing Sci-Hub in the Indian courts, and will probably be successful. (While that suit continues, Sci-Hub has agreed to not upload any new articles and books, so there is already some success.) • The bullet point above is a specific example of a more general problem in the paper – while the removal (or even reduction) of copyright will bring great benefit, it will also have drastic negative consequences, and the authors have not raised these at all. For an opinion piece to have validity, opposing positions do need to be considered.• I would suggest that Wikipedia is not a good source to cite in an academic journal (in fact, Jimmy Wales has said as much). It would be far preferable for the authors to trace the sources of the information, and cite those sources).• Along these lines, it would be better to refer to Richard Stallman by name (and cite his work), rather than refer to him as merely “a programmer”. (And it might be a good idea to note that the term copyleft had been used as early as 1976 by Li-Chen Wang, and to cite that source also.)• An area that the authors really do need to correct is the language. The paper suffers from a large number of language errors. Most of them are minor, irritating, but some are grave enough to interfere with the message. (Sometimes, the error may be language, or may be simply incorrect, so a statement like “The societies realized that this pandemic is first in digital era” can only be taken as a very vague statement, and is not easily supported – during the digital era, there have been several pandemics, although none have had such a widespread impact as COVID-19). Many sentences require re-reading, and I would warrant that many readers would simply not make the effort to complete reading the paper. I would suggest that Version 2 of the paper could benefit from a very careful and close proof-read before submission.So, overall, I think that the paper has a kernel of an argument, which appears to be that the events surrounding COVID-19 should give us pause to consider the negative impact of copyright restrictions on medical education, and that, after the pandemic, we should continue with efforts to make medical information more easily accessible, particularly through a reduction of these copyright restrictions. While there are weaknesses to the argument, the problem is that that the paper does not express this very well. I look forward to Version 2 of the paper in which the issues I have raised are addressed.

Reviewer Expertise:

NA

No decision status is available

# Reviewer report: Reviewer response for version 1

**DOI:** 10.21956/mep.19014.r27286

**Published:** 2021-02-02

**Authors:** Keith Wilson

**Affiliations:** 1 Dalhousie University

This review has been migrated. The reviewer awarded 2 stars out of 5 The authors of this opinion piece make the case for sustaining the momentum towards a more democratized medical education system worldwide. They draw on numerous examples that emerged during the COVID pandemic including the breaking down of traditional barriers to educational materials and other resources.One of the most important aspects of the paper is its highlighting of the challenges that are faced in limited resource settings. This forms the foundation for making the case to share resources more uniformly. The authors use the term “democratization of medical education” in numerous contexts within the paper. I feel that readers would be very appreciative of a framing definition located early in the paper to help orient the reader to the rest of the arguments being put forth.As suggested by the title by the title of this paper, the authors illustrate the differences between copyright, copyleft and a more recent term copycovid. The progression of these concepts could use some expansion of the explanation of how they are interrelated. In particular, examples were given that highlight a number of the strides that occurred during the pandemic to improve access. This section of the paper (“Time Changes Things”) would benefit from some restructuring to group the ideas together (e.g. under organizational, educational, human capital, etc. headings). The narrative would benefit from a more linear flow of the ideas to reach the conclusion. There are so many excellent observations highlighted in the paper – to this end, it would be very beneficial to group them more clearly. I worry that without more structure, the astute messages that the authors are trying to convey may get lost. I would recommend some reworking of the flow to make the conclusions more compelling for the reader.The authors of this paper cite many good examples and insights that support their argument for the notion that everyone benefits from resources being distributed more equally. They are to be commended for bringing together a number of different observations on how COVID has reshaped the way we develop educational opportunities now and for the future.

Reviewer Expertise:

NA

No decision status is available
